# Sustainable Magnetochromic Flexible Composites for Advanced Optical Sensing and Smart Applications

**DOI:** 10.1021/acsaenm.4c00774

**Published:** 2025-04-11

**Authors:** Rita Polícia, Ricardo Brito-Pereira, Bruna F. Gonçalves, Pedro Martins, Senentxu Lanceros-Méndez

**Affiliations:** 1 Physics Centre of Minho and Porto Universities (CF-UM-UP) and Laboratory of Physics for Materials and Emergent Technologies, LapMET, 56059 University of Minho, Braga 4710-053, Portugal; 2 Centre of Chemistry, University of Minho, Braga 4710-053, Portugal; 3 BCMaterials-Basque Center for Materials, Applications and Nanostructures UPV/EHU Science Park, Leioa 48940, Spain; 4 Ikerbasque, Basque Foundation for Science, Bilbao 48009, Spain

**Keywords:** sustainable materials, magnetochromism, flexible electronics, optical sensors, nanostructured materials

## Abstract

This study presents the development of sustainable, flexible magnetochromic (MC) materials using Fe_3_O_4_ (FO) and CoFe_2_O_4_ (CFO) nanoparticles synthesized via environmentally friendly methods, along with NdFeB (ND) swarf from neodymium–iron–boron magnet waste. These nanoparticles are integrated with thermochromic (TC) pigments and poly­(vinyl alcohol) (PVA), forming a transparent, flexible polymer matrix. The use of green synthesis for FO and CFO minimizes hazardous chemicals and energy consumption, while the recycling of ND swarf addresses waste management and provides a cost-effective magnetic material. The resulting MC materials can be printed using eco-friendly techniques, offering a sustainable alternative to traditional sensing materials. The devices demonstrate fast color changes between red and gray within 3–5 s under an alternating magnetic field (200 Oe, 100 kHz), making them ideal for applications in flexible electronics, smart textiles, and sensors. With a focus on environmental responsibility, these materials meet the increasing demand for sustainable technologies in advanced optical sensing systems.

## Graphical abstract

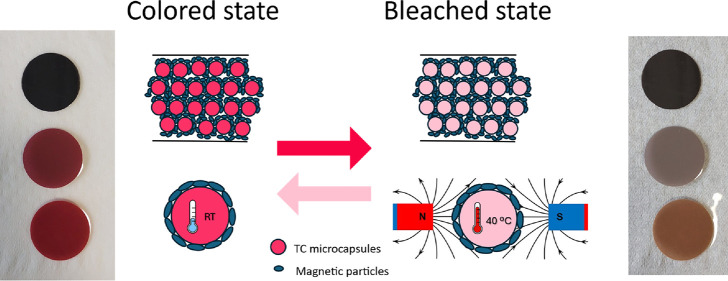

## 1 Introduction

In today’s rapidly advancing technological landscape, the demand for innovative materials that offer dynamic and versatile functionalities is higher than ever before.^1,2^ Magnetochromic (MC) smart materials, with their ability to reversibly change optical properties in response to magnetic fields, represent a cutting-edge solution with strong potential in a large variety of applications.^3–5^ This unique property has garnered considerable interest across various scientific and technological fields due to benefits such as contactless control, instant action, and ease of integration into electronic devices.^5–7^ These advantages have led to the exploration of MC applications in diverse areas, including sensing, imaging, adaptive optics, displays, optical switches, security, and anticounterfeiting.^8–10^

Recent studies on MC materials have focused on developing colloidal photonic nanocrystals with tunable photonic properties in the visible spectrum. These nanocrystals can rapidly and reversibly change color in response to an external magnetic field due to alterations in their spatial arrangement or magnetic interactions.^11,12^ However, the colloidal stability of such systems is poor due to chain aggregation of dynamically ordered assemblies induced by the magnetic packing force.^13,14^ To overcome this issue, a polymer matrix has been used to stabilize the photonic structures and consequently the diffraction color.^8,15–17^ Nevertheless, the magnetic field-responsive chromic capability decreased since the ordered array structure is fixed inside the polymer.^13^ In a recent study, MC materials have been developed based on an elastomer composed of PDMS and Fe_3_O_4_ (FO) nanoparticles. Under an external magnetic field, the nanoparticles form rod-like structures that align with the field, allowing light to pass through the elastomer and change color from dark gray to transparent/light gray in 30 s.^18^ Although these materials exhibit good optical densities, two main problems limit their practical applications: (i) the dark color of the magnetic particles, which restricts color change to dark-to-transparent/light gray, and (ii) the response time, which remains in the tens of seconds, insufficient to compete with other color-changing technologies that can alter color in a few seconds. Alternatively, composites with an extrinsic MC effect, combining the magnetothermal and thermochromic effects, have emerged as a novel approach for a new generation of color displays. As a good example of this strategy, Wang et al. developed a solid magnetic photonic hydrogel based on FO@SiO_2_ colloids embedded in a copolymer hydrogel of *N*-isopropylacrylamide (NIPAM, as a thermosensitive monomer). The MC effect was produced under an alternating magnetic field, changing color from green to blue in 6 min.^13^

Beyond the stability and performance of the materials, the scalability and sustainability of the fabrication method are critical for reaching practical applications.^19,20^ Additive manufacturing methods have gained attention due to their ability to produce cost-effective and scalable systems in a variety of shapes and sizes.^21^ Unlike traditional manufacturing methods, which rely on subtractive processes or masking techniques like etching or deposition, printed smart materials use additive processes to build lightweight electronic components on rigid and flexible substrates.^22^ As a result of this scenario, printed MC smart materials have been developed. A novel printable MC ink based on photonic granular hydrogel for customized structural colored objects has been obtained exhibiting shear thinning and self-healing, enabling direct writing of macroscopic structural colored patterns by 3D printing.^23^ This work demonstrates the versatility of MC materials and their capability of integration into current fabrication methods. However, despite this progress toward eco-friendliness using sustainable fabrication methods, devices should be built with source-efficient materials to ensure minimal environmental impact throughout their entire life cycle, from manufacturing to disposal. In this technological MC arena, we propose novel sustainable MC inks based on three different magnetic structures: FO nanoparticles and CoFe_2_O_4_ (CFO) nanoparticles that can be produced by eco-friendly green synthesis,^24,25^ and NdFeB (ND) swarf that can be obtained from neodymium–iron–boron magnet waste.^26^ These magnetic structures are able to generate heat when exposed to magnetic fields^27–29^ and, when combined with thermochromic (TC) pigment^30^ and poly­(vinyl alcohol) (PVA) as a flexible, transparent, and sustainable polymer binder,^31^ allow the production indirect MC effect. The PVA polymer binder also confers mechanical integrity to the final composite, allowing it to be processed by screen-printing techniques, and adding good chemical and mechanical stability.^32^ Its hydrophilic nature also promotes a uniform distribution of the magnetic particles, guaranteeing an efficient response to external magnetic fields.^33^ The heat generated from the magnetic particles, due to the applied magnetic field, triggers a phase change in the TC pigments, causing the material to change color. Among the TC materials, reversible TC microcapsules are a mature technology that has been extensively applied in solar energy storage, wearable electronic devices, smart textiles, medical diagnostics, smart coatings, and inks.^34–36^ Leuco-dye-based reversible TC compositions are recognized as one of the most promising organic TC materials owing to their high sensitivity to temperature changes, as well as low cost, chemical stability, and high durability.^37^ Consequently, these materials were used as core materials for fabricating composite inks with magnetic/thermal-sensitive properties.

The inks were printed on a PVA substrate film, forming a magnetic sensor/display that can be easily recycled through dissolution in water and magnetic separation.

## 2 Materials and Methods

### 2.1 Materials

Poly­(vinyl alcohol) (PVA), 98% hydrolyzed with Mw of 13,000–23,000, was purchased from Sigma-Aldrich (Missouri, USA). Neodymium (ND) particles with a 5 μm particle size were purchased from Magnequench (Chuzhou, China). CoFe_2_O_4_ (CFO ≈ 30–55 nm) and Fe_3_O_4_ (≈30 nm) nanoparticles were from NanoAmor. TC powder with a reversible TC effect, changing from red to translucent from 31 °C (Thermochromic Pigment Red 31 °C), was purchased from SFXC Good Life Innovations Ltd. (Denton Island, UK), and distilled water was prepared in our laboratory. All materials were used as received from the providers.

### 2.2 PVA-Based Substrate Preparation

For the preparation of the substrate, 1 g of PVA powder was added to 6 mL of distilled water and placed under mechanical agitation until complete dissolution of the polymer. Afterward, the solution was homogeneously spread over a glass substrate using a doctor blade with a 480 μm spacer. After the spreading step, the samples were left to dry overnight until the complete removal of the solvent. Then, the ≈0.1 mm-thick films were removed from the glass substrate.

### 2.3 PVA/MC Ink Preparation

To test the influence of magnetic particle content on the MC behavior, CFO particles were added in different concentrations (20, 33, and 40 wt %) to 6 mL of distilled water. The particles were dispersed in an ultrasound bath (Fisherbrand FB15056) for 2.5 h to ensure good dispersion and prevent particle agglomeration. Furthermore, dispersions with 40 wt % of ND and FO were also produced in the same way to assess the influence of particle type on the MC response. Then, PVA powder was added to the solution under mechanical stirring (Haidolph Instruments, RZR 1) using a PTFE stirrer until complete dissolution of the polymer at 90 °C. Finally, the TC powders were added in concentrations of 40, 33, and 20 wt % and mechanically dispersed for 1 h at room temperature. The resulting solution was homogeneously screen printed over the PVA substrates, using a polyester mesh with 100 threads cm^–1^ reaching a final thickness of 14.5 μm.

After spreading, the samples were left overnight at room temperature, to ensure complete water evaporation.

### 2.4 Characterization Methods

Rheological properties were assessed at room temperature using an AresG2 rheometer, featuring a flat plate configuration with a 40 mm diameter. Flow curves were generated by maintaining a 500 μm gap between the plates and applying a linear shear rate ranging from 0.1 s^–1^ to 300 s^–1^. The dynamic viscosity values (μ) were calculated from the shear stress (τ) as a function of shear rate $\left(\frac{du}{dy}\right)$ , as shown in the inset of the Figure 2a, using the Newton’s law of fluids:

$$\tau =\mu \times \frac{du}{dy}$$ 1

Surface morphology was examined using scanning electron microscopy (SEM) with a JEOL JSM-7000F equipment operating at 10 kV, on samples coated with a ∼15 nm-thick conductive gold layer (Quorum Q150TS turbo-pumped coater). Elemental analysis was conducted via energy-dispersive spectroscopy (EDS) with an EDX Oxford Instruments system at 10 kV.

Attenuated total reflection-Fourier transform infrared (ATR-FTIR) spectroscopy was performed using a Jasco FT/IR-6100 system in the spectral range of 4000 to 600 cm^–1^, with 64 scans at a resolution of 1 cm^–1^.

Mechanical stress–strain properties were measured in tensile mode using a TST350 Linkam Instrument (Tadworth, England), at a strain rate of 100 μm/s. Samples with dimensions of 5 × 15 × 0.1 mm were tested in triplicate. The mechanical values are provided as the average of those measurements. The Young’s modulus of the materials was determined in the linear region within the range of 0 to 2% deformation.

The adhesion of the printed inks to the substrate was evaluated using a modified tape peel test on samples measuring 1 cm × 1 cm. An adhesive tape (3 M Scotch Magic Tape 810) was applied to the surface of the printed samples with varying forces (50, 100, 200, and 300 N) for 30 s. This was achieved using a Shimadzu AG-IS universal testing machine in compression mode at a speed of 2 mm min^–1^. After applying the tape, it was peeled off at the same speed while the force exerted on the sample was recorded. The samples were weighed before and after each test to obtain the mass loss in the printed layer.

Thermal properties were analyzed by thermogravimetric analysis (TGA) using an NETZSCH STA 449F3 DSC-TGA equipment. Samples were heated from 35 to 700 °C at a rate of 20 °C min^–1^ under a nitrogen atmosphere. Differential scanning calorimetry (DSC) was performed using a DSC3+Mettler Toledo instrument, by placing the samples in standard sealed alumina pans and heating them from 25 to 250 °C at 20 °C min^–1^ in a nitrogen gas flow rate of 50 mL min^–1^.

Magnetic hysteresis loops at room temperature were measured using a vibrating sample magnetometer (Oxford Instruments) up to a maximum field of 1.8 T.

The chromaticity analysis of the MC materials was carried out before and after heating the samples at 40 °C in fiber-optic spectrometer AvaSpec-ULS2048CL-EVO connected to fiber-optic cable FCR-7UVIR200-2-ME (Avantes) placed at a 45° angle with respect to the sample’s surface. The AC magnetic field for the MC characterization was provided by an AEG HK874400FB Pure 4 induction system (0–50 mT, 20–80 kHz).

## 3 Results and Discussion

### 3.1 Morphological, Mechanical, and Physicochemical Characterization

Morphological characterization of the neat PVA substrate and the corresponding printed layers of each of the developed MC inks was performed by SEM. In Figure 1, cross-section images with the corresponding EDS analysis (Figure 1a–u) and surface images (Figure 1v–ai) of the different samples are presented.

**Figure 1:**
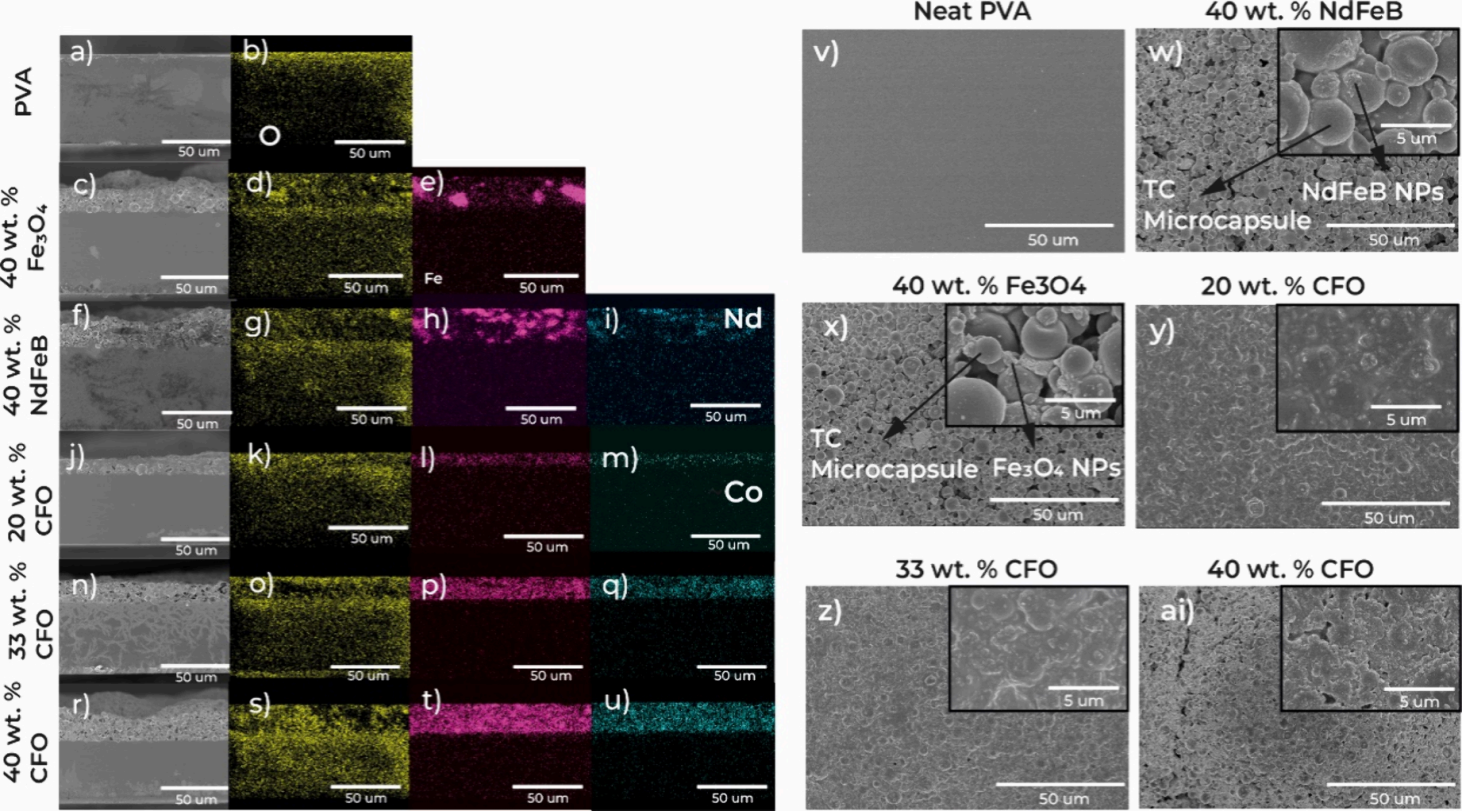
SEM cross-section images of (a) neat PVA and printed MC inks based on PVA, TC microcapsules, and different magnetic nanoparticles: (c) 40 wt % Fe_3_O_4_, (f) 40 wt % ND, and CFO at concentrations of (j) 20 wt %, (n) 33 wt %, and (r) 40 wt %. Corresponding EDS mapping images for O (oxygen, yellow: b, d, g, k, o, and s), Fe (iron, pink: e, h, l, p, and t), Nd (neodymium, blue: i), and Co (cobalt, green: m, q, and u) atoms. Surface morphology of (v) neat PVA and respective composites based on (w) PVA/40 wt % ND/40 wt % TC NPs, (x) PVA/40 wt % FO/40 wt % TC NPs, (y) PVA/20 wt % CFO/40 wt % TC NPs, (z) PVA/33 wt % CFO/33 wt % TC NPs, and (ai) PVA/40 wt % CFO/40 wt % TC microcapsules. Inset: amplified surface images of the composites on a scale of 5 μm.

The pristine PVA substrate shows a smooth and compact morphology both in cross section and surface (Figure 1a,v), with no visible cracks or voids.

In Figure 1j–u, the increase in the magnetic particle concentration and consequently the decrease of polymer content led to the increase in the layer thickness from ≈8.3 μm for 20 wt % of CFO, to ≈14.3 μm for 33 wt % of CFO, and to 18.4 μm for 40 wt % of CFO.

SEM surface images of the MC layers with increasing CFO content from 20 to 40 wt % are presented in Figure 1y–ai. As the magnetic particles are nanosized, the difference in the morphology of the composites’ surface mainly relies on the ratio between TC microcapsules and PVA. For the samples with 20 and 33 wt % CFO, the TC/polymer ratio is 1. Consequently, they present similar smooth morphology. However, on an amplified scale (inset images of Figure 1y,z), it can be seen that the higher number of CFO nanoparticles induces greater roughness in the surface morphology.

Both the cross-sectional and surface images presented in Figure 1 show that the majority of the microcapsules did not collapse as a result of the shear stress induced by the screen-printing process, indicating that these TC pigments are well suited for screen-printing applications.

CFO particles are uniformly dispersed independently of the concentration used; therefore, MC inks with ND and FO were fabricated using 40 wt % in order to optimize the MC performance.

In the cross-section images of 40 wt % ND and 40 wt % FO (Figure 1c–i), it is possible to distinguish the MC layers printed in the PVA substrate with a thickness of ≈25 μm. EDS distribution of the Fe and Nd elements (Figure 1e,h,i) shows small agglomerates in the functional layer, in particular in the case of ND. These agglomerates might contribute to a decrease in the MC effect compared to the CFO-based MC materials, where the particles are more uniformly dispersed (Figure 1m,q,u).

The surface images of the 40 wt % ND (Figure 1w) and 40 wt % FO (Figure 1x) display a uniform distribution of the TC microcapsules with spherical morphology and sizes ranging from 1 to 5 μm, observed in the corresponding inset images. The magnetic nanoparticles are also visible in the inset images, appearing as small agglomerates around the TC microcapsules.

The morphology of the sample with 40 wt % of CFO is similar to the ones with 40 wt % of FO and ND, since the TC microcapsule/PVA ratio is the same, equal to 2. These results demonstrate that the morphological and compositional properties of the printed MC layers, including particle distribution and layer thickness, are relevant parameters for optimizing the morphological and functional characteristics of the MC materials. Uniform particle distribution ensures consistent optical properties, while the appropriate layer thickness and minimal agglomeration are essential for effective color change response and material durability.

Determining the viscosity of the processed inks is critical for their suitability for being applied by specific printing technologies. For the screen-printing technique, suitable viscosity values range from 0.1 to 100 Pa·s.^38^

The ink composites based on 40 wt % of ND and 40 wt % FO exhibit the highest viscosities at higher shear rates, with values of 3.02 Pa·s, followed by 40 wt % of CFO and 20 wt % of CFO, with 0.91 Pa·s, and 33 wt % of CFO ink with 0.23 Pa·s. For the minimum applied shear rate of 1.62 s^–1^, the inks based on 40 wt % of ND and 40 wt % FO present viscosities of 10.31 and 11.73 Pa·s, respectively, which rapidly decrease with the increase of the shear rate up to 100 s^–1^. The higher viscosity of inks containing ND and FO particles at high shear rates, compared to inks with CFO particles, can be attributed to differences in particle size. Smaller particles such as CFO lead to a more homogeneous dispersion, as observed in the SEM images (Figure 1), while larger particles as ND and FO cause increased friction and resistance to flow, leading to higher viscosity.

The viscosity of the inks containing CFO increased with the increase of magnetic particle concentration for low values of shear rate due to several factors, including particle interaction, microstructural changes, and magnetic effects. With the increase of the CFO concentration, van der Waals forces and magnetic interactions increase, creating a more interconnected network within the ink, which resists flow and increases viscosity. Therefore, increasing the shear rate will disperse and increase the space between particles, which will reduce their interactions and, consequently, reduce their viscosity.^39^ This phenomenon explains the more significant viscosity drop observed with increasing shear rate for higher concentrations of CFO.

In the ink formulation containing 33% CFO, both the PVA and TC microcapsules' concentration is 33 wt %, while in the other inks, the concentration of TC microcapsules was kept constant at 40 wt %. Therefore, the ink containing 33 wt % of CFO allows the evaluation of the effect of decreasing the TC particles’ concentration. As observed, at low shear rates, the viscosity is primarily influenced by the particle content. However, as the shear rate increases, the viscosity decreases more significantly (0.23 Pa·s) in the ink containing 33 wt % CFO than in the inks containing 20 and 40 wt % CFO (0.91 Pa·s). This suggests that at higher shear rates, the polymer concentration plays a more dominant role in determining the viscosity. Most importantly, the viscosity of the developed inks ranges between 0.23 and 30 Pa·s ([Fig fig2]a), which is in the suitable range of viscosities for screen-printing inks. Moreover, the inks exhibit pseudoplastic (shear thinning) behavior, characterized by a reduction in viscosity with an increasing shear rate until a minimum viscosity is achieved, beyond which the viscosity stabilizes at a constant value. This behavior is suitable for screen-printing inks and pastes since it provides good ink transfer.^40^

**Figure 2:**
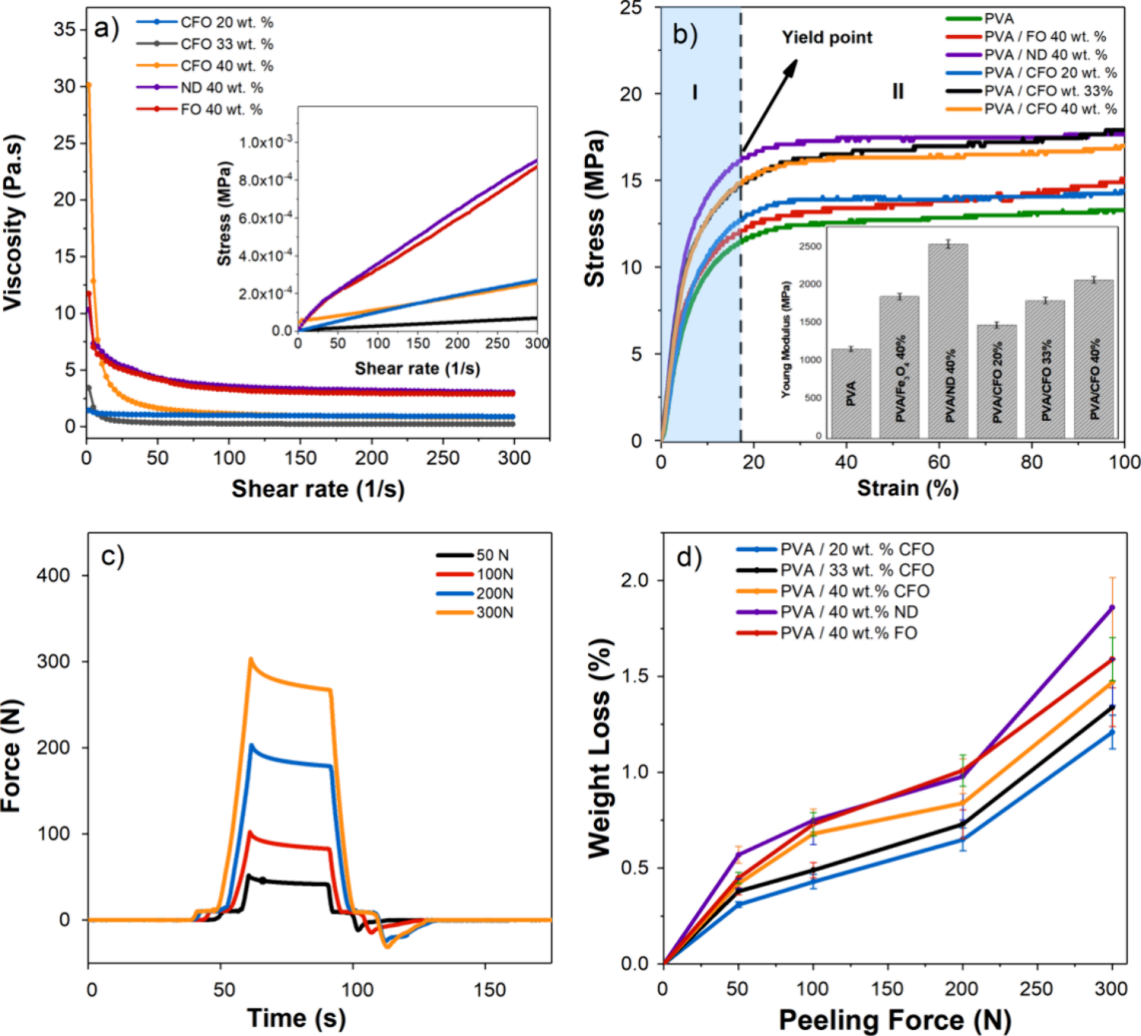
(a) Viscosity as a function of shear rate at 25 °C for different composite inks: PVA/40 wt % ND/40 wt % TC NPs; PVA/40 wt % FO/40 wt % TC NPs; PVA/20 wt % CFO/40 wt % of TC NPs; PVA/33 wt % CFO/33 wt % of TC NPs; and PVA/40 wt % CFO/40 wt % of TC NPs. Inset: shear stress as a function of shear rate. (b) Stress–strain curves of different PVA-based inks, namely, PVA; PVA/FO 40 wt %; PVA/ND wt. 40%; PVA/CFO (20, 33, and 40 wt %), with an inset showing the mean Young’s modulus of the samples, determined for deformations up to 2%. (c) Force profile of the peeling assays for the PVA/40 wt % CFO/40 wt % TC microcapsule sample as a function of time under different applied compression forces. (d) Weight loss of the printed MC inks after the peeling assays as a function of the compression force.

After printing the MC inks on neat PVA substrates using the developed inks, the mechanical properties of the systems were assessed (Figure 2b). The stress–strain curves of neat PVA and PVA substrates with a single printed layer of different MC inks follow the tendency of a thermoplastic polymer that includes an initial elastic region (region I) with a linear increase in stress with strain, followed by yielding, and then a plastic deformation region (region II) where the material undergoes significant elongation at relatively constant stress before finally reaching failure or fracture. As observed in Figure 2b, the printed layer has a determining effect on the mechanical properties of the samples, increasing the Young’s modulus of the structure.^41^ Specifically, the observed Young’s modulus of 1185 ± 31 MPa for neat PVA is enhanced to 2572 ± 55 MPa for the PVA/ND 40 wt % sample, 1878 ± 42 MPa for PVA/FO 40 wt %, and 2098 ± 43 MPa for the PVA/CFO 40 wt % sample. The increase in Young’s modulus resulting from the addition of magnetostrictive fillers can be attributed to two factors. First, there is a mechanical enrichment that arises from the surface interaction between the magnetic particles and the PVA chains. Second, the higher Young’s modulus of the fillers themselves (CFO ≈ 150 GPa, TD ≈ 50–90 GPa, and ND ≈ 45 GPa) also contributes to the overall increase,^42^ this increase being proportional to the particle content, in the case of CFO.

The variations in Young’s modulus of the ND-containing inks are thus attributed to the superior mechanical reinforcement ability of the microsized ND due to stronger physical interactions, namely, electrostatic and van der Waals interactions, between the polymer matrix and fillers, leading to uniform stress distribution along the composite, improved interfaces, and the absence of voids at the interface.^43,44^

The adhesion between the PVA substrate and the layer of MC ink was studied by an adapted tape peel test.^44^ Four different compressive forces of 50, 100, 200, and 300 N were applied in the perpendicular direction to the substrate, after which the force was removed, and the peeling force was measured (Figure 2c). The amount of printed material detached from the substrate upon these tests was determined in weight loss percentage (Figure 2d). According to Figure 2c, there is a direct relationship between the peeling force and the compression force. Specifically, the peeling force is found to be maximum when the compression force is 300 N, resulting in a peeling force of 15.1 N. Conversely, the lowest peeling force is seen when the compression force is 50 N, leading to a peeling force of 2.2 N. The mass loss obtained in all samples is directly proportional to the force exerted by the load cell, as depicted in Figure 2d. The results show that the amount of weight loss increases with the increase of the magnetic particle concentration, where the PVA/ND 40 wt % sample demonstrates the greatest mass reduction (≈1.8%) in comparison to inks containing CFO (≈1.5%) and FO (≈1.6%). This small variation may be attributed to the larger diameters of neodymium followed by the FO particles, which can result in more irregular surfaces, form aggregates due to particle–particle interactions, and, consequently, reduce ink adhesion. Conversely, the ink containing 20 wt % CFO exhibits the lowest weight loss (≈1.2%) at 300 N of applied force, aligning with existing literature that suggests a slight decrease in ink adhesion to the substrate with increasing filler concentration from ≈1.2% for 20 wt % CFO to ≈1.3% for 33 wt % CFO, and ≈1.5% for 40 wt % CFO.^45^ The ink with the reduced ratio particles/polymer (33 wt % CFO) follows the weight loss tendency of the other samples, which indicates that the amount, size, and distribution of the magnetic particles are determinant factors for the mass loss during peeling. In the subsequent iteration of experiments, it was observed that none of the specimens exhibited a substantial reduction in weight (below 0.1%), irrespective of the specific ink used. The values mentioned exhibit a favorable comparison with those documented in the literature on the effective printing of metal nanoparticle inks onto polymeric surfaces ^1^.

DSC analysis revealed distinct thermal transitions in PVA-based composites incorporating TC pigments and different concentrations of magnetic nanoparticles (FO, ND, and CFO) (Figure 3a).

**Figure 3:**
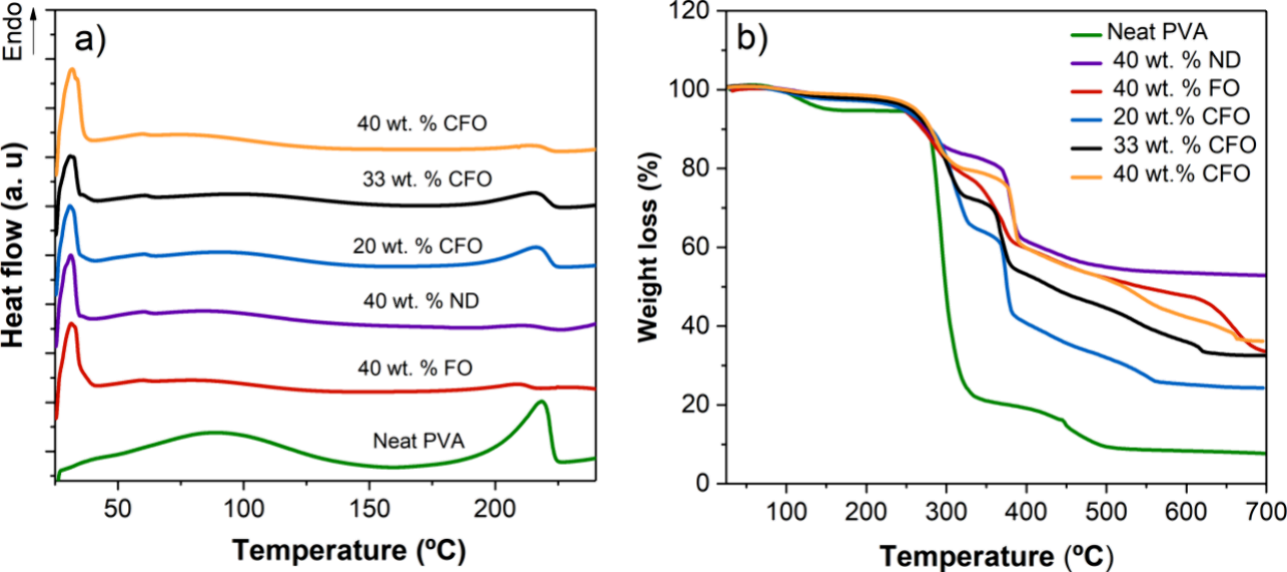
(a) Differential scanning calorimetry (DSC) heating thermogram and (b) thermogravimetric of neat PVA and respective MC composites with different concentrations of magnetic particles.

An endothermic peak is observed around 30 °C across all samples that contain TC pigments, which is indicative of the microcapsules’ phase change that induces the TC effect. Regarding the pristine PVA sample, a pronounced broad endothermic peak is observed around 230 °C, corresponding to the polymer’s melting temperature.^46^ In relation to the composite samples, the decrease in PVA content results in a less prominent endothermal peak.^47^ It is observed that the peak intensity increases proportionally with the PVA concentration in all samples, which confirms the linear relation of this peak with the PVA melting.^26,48^

TGA presents the mass loss behavior of PVA-based composites (Figure 3b). These results indicate that the degradation of pristine PVA inks is characterized by three distinct weight loss phases, as documented in the literature.^48^ The first phase takes place at approximately 100 °C and is associated with the evaporation of the remaining water from the ink. The majority of weight loss occurs at approximately 300 °C, primarily due to the removal of the lateral group acetate. This phenomenon is followed by a subsequent weight loss between 400 and 450 °C, which corresponds to the degradation rate of the PVA polymeric main chain.^48,49^

Regarding the composite inks, it is observed that the TC pigments present a thermal decomposition that starts to occur at 300 °C, and at 500 °C they undergo the total degradation, which is in accordance with the manufacturer’s technical data.^50^ The inks with CFO nanoparticles present an additional weight loss of 5% between 400 and 600 °C, which is accounted for by the decomposition of organic compounds present in the CFO nanoparticles.^51^ The inks with the ND nanoparticles present a different thermal behavior at higher temperatures, above 400 °C, due to the properties of ND. These nanoparticles begin to gain weight continuously with the increase of temperature due to the formation of various oxides while the oxidative attack proceeds from the Nd-rich grain boundary phase.^52^

Finally, the TGA reveals that the incorporation of different nanoparticles and TC pigments in PVA matrices affects overall thermal decomposition behavior. Composites with higher nanoparticle content exhibit improved thermal stability, as evidenced by the delayed onset of decomposition and slower weight loss rates. The final residue analysis also confirms the presence and stability of inorganic nanoparticles at higher temperatures. Since the magnetic properties of the MC composites have a critical impact on their functional performance, the corresponding room-temperature responses have been evaluated as a function of the filler type and content (Figure 4).

**Figure 4:**
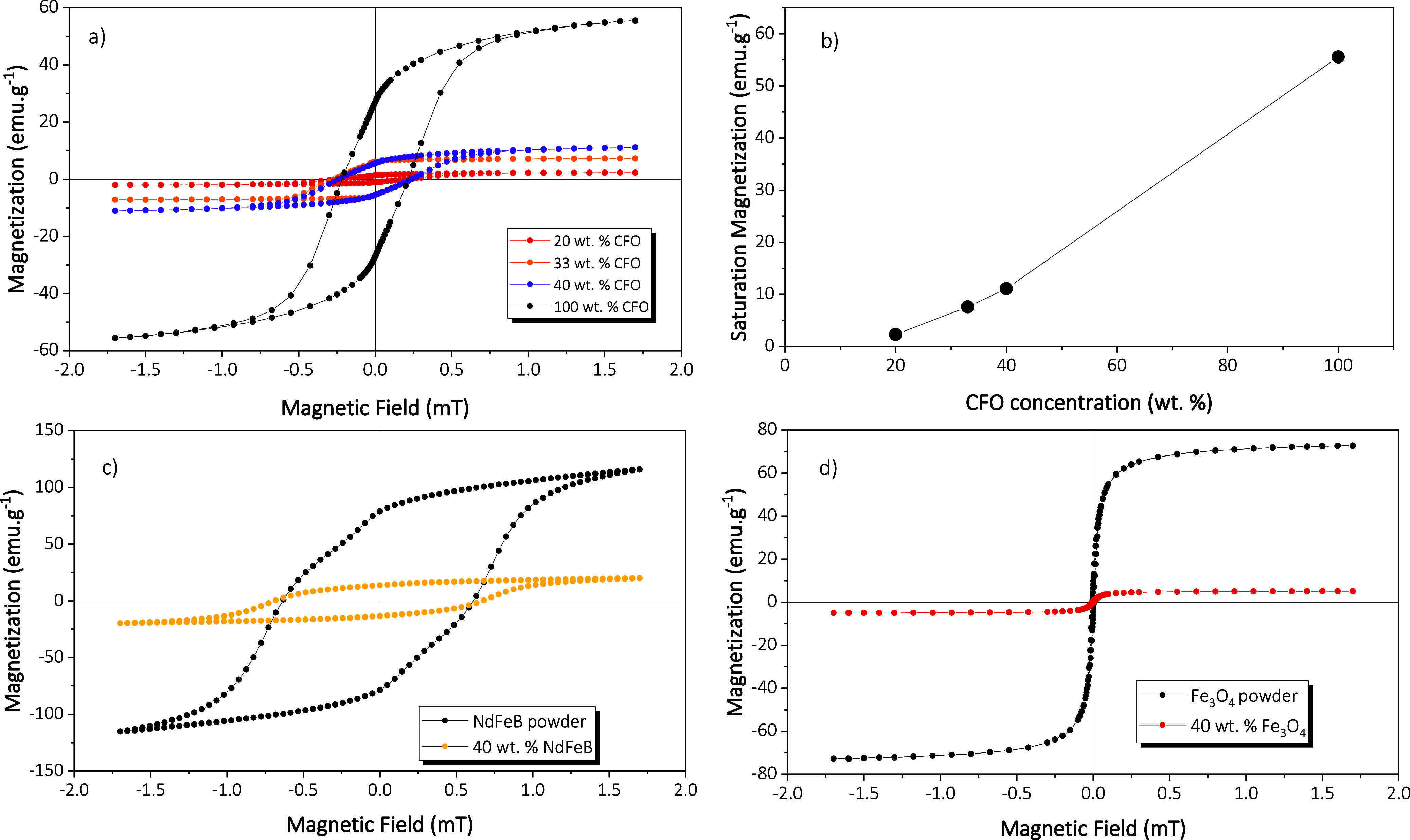
(a) Room-temperature hysteresis loops for pure CFO nanoparticle powder and for PVA/TC microcapsule-based MC inks with 20, 33, and 40 wt % of CFO nanoparticles. (b) Saturation magnetization as a function of CFO nanoparticle concentration. Room-temperature hysteresis loops for (c) pure ND nanoparticle powder and PVA/TC microcapsules/ND-printed MC inks and (d) pure FO nanoparticle powder and PVA/TC microcapsules/FO-printed MC inks.

Hysteresis loops of the ME composites reveal the distinct magnetic behaviors (Figure 4a,c,d) induced by the different nanoparticles: CFO-based composites develop hysteresis loops with coercivity of 0.24 mT and remanence of 1.35, 5.68, and 6.16 emu g^–1^, for CFO concentrations of 20, 33, and 40 wt %, respectively; ND composites exhibit higher coercivity (≈0.68 mT) and remanence (≈13.69 emu g^–1^); and FO-based composites show a complete absence of hysteresis, remanence, and coercivity. The observed behaviors are compatible with ferromagnetism in CFO-based and ND-based composites, and superparamagnetism in FO-based composites. In the FO case, room temperature is above the blocking temperature, and the magnetic moment of the particle is free to rotate in response to the applied magnetic field.^53^

The shape and maximum magnetization values of the measured hysteresis loops for the composite samples, shown in Figure 4a, demonstrate that magnetic particles are randomly oriented within the polymer matrix. Additionally, the maximum magnetization values of 2.27, 7.26, and 11.08 emu·g^–1^ found for 20 wt % CFO, 33 wt % CFO, and 40 wt % composites, respectively, reveal that the nanoparticles are well distributed and dispersed (Figure 4b). Furthermore, the maximum magnetization value is directly proportional to the amount of nanoparticles inside the polymer-based composite (the saturation magnetization of pure CFO nanoparticles is 55.4 emu g^–1^). Such proportionality is also observed in the saturation magnetization value of 40 wt % ND composites shown in Figure 4c, which is 19.9 emu g^–1^, and the saturation magnetization of pure ND nanoparticles is 115.8 emu g^–1^. For 40 wt % FO composites (Figure 4d), the saturation magnetization value is 5.05 emu g^–1^ and the saturation magnetization of neat FO nanoparticles is 72.8 emu g^–1^.

### 3.2 Magnetochromic Functional Response

After the structural, mechanical, adhesion, thermal, and magnetic properties have been validated, the MC properties have been evaluated (Figure 5). Figure 5a displays a schematic representation of the MC working mechanism of the materials and photographic pictures of the printed MC inks containing 40 wt % of CFO, FO, and ND (from up to bottom) at room temperature (bleached state) and at 40 °C (colored state) to induce the maximum color change of the TC microspheres. To study the MC functional response, the samples were subjected to an alternating magnetic field ranging from 0 to 50 mT at a frequency of 40 kHz. Upon the application of an alternating magnetic field, the magnetic nanoparticles produce heat due to the hyperthermic effect, increasing the temperature of the surrounding polymer matrix and the embedded TC microcapsules. As the temperature of the microcapsules reaches the activation threshold, the encapsulated TC pigment undergoes a reversible chemical change, resulting in a visible color transition from red to translucent. This change in the TC pigment causes the overall composite to shift from a red shade to the characteristic color of each of the magnetic particles, as can be seen in the photographic picture of Figure 5a. Upon removal of the magnetic field, the magnetic particles cool down, the heat dissipates, and the temperature of the microcapsules decreases, reversing the TC color change, consequently returning the ink to its original state.

**Figure 5:**
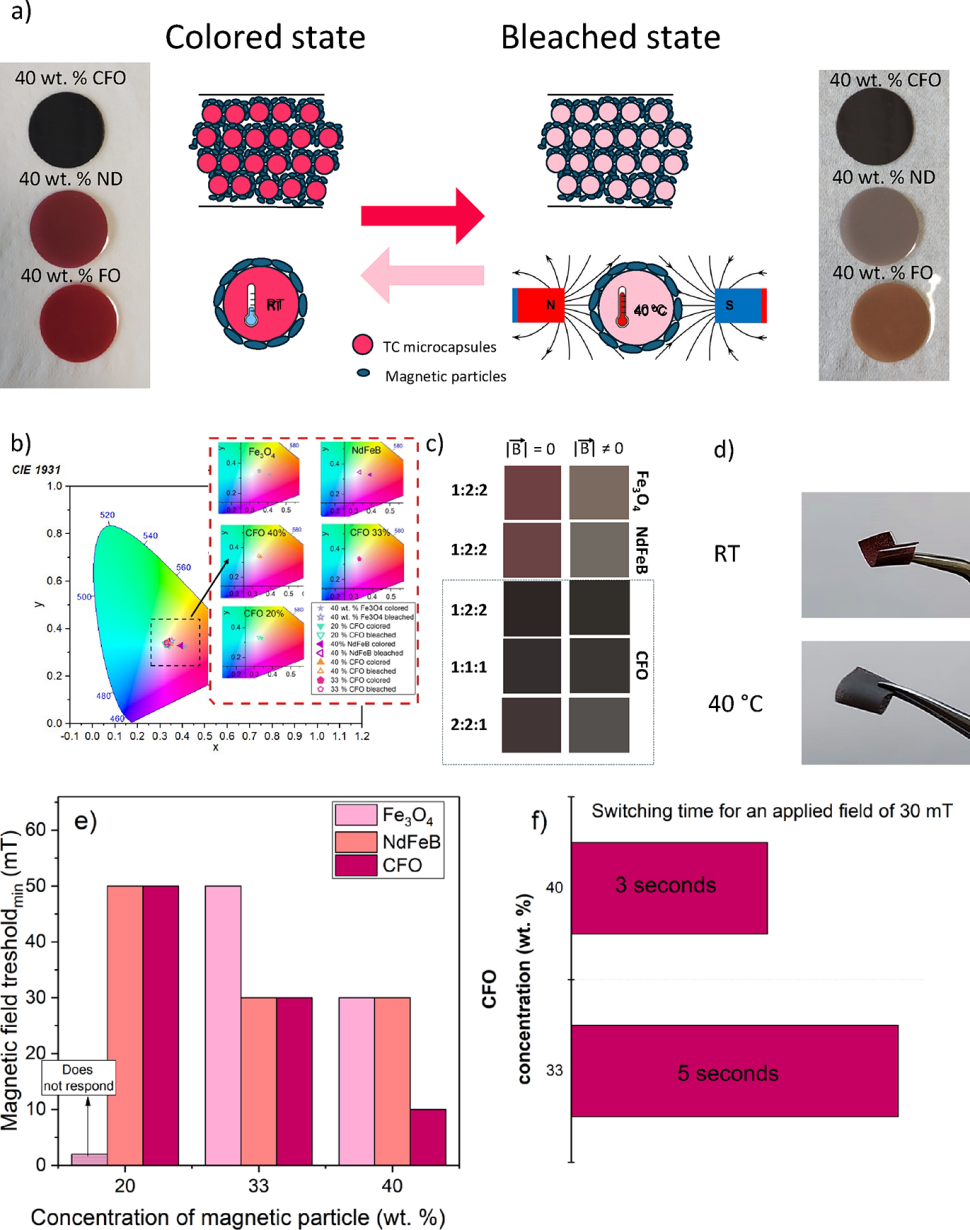
(a) Schematic mechanism of the color-changing response of the composite to a magnetic field through a hyperthermia/thermochromic coupled effect, along with photographic images of the printed composites based on 40 wt % of CFO, ND, and FO (in descending order) on a PVA substrate at room temperature (left images) and at 40 °C (right images). (b) CIE coordinates showing the composite differences in chroma values before and after applying magnetic field, and (c) corresponding RGB color according to the polymer/TC particles/magnetic particle ratio. (d) Images of the printed MC composite with 40 wt % ND being bent at room temperature (upper image) and at 40 °C (lower image). (e) Minimum magnetic field magnitude required to change the composite’s color as a function of magnetic particle concentration. (f) Switching times of CFO-based MC composites as a function of CFO concentration.

The significant difference in the optical responses can be visualized as CIE (Commission Internationale de l’Eclairage) coordinates placed in the 1931 color space diagram (Figure 5b). The inset images of Figure 5b show the difference in CIE coordinates for each MC ink on their bleached and colored states. These coordinates were also converted into RGB colors, as shown in Figure 5c, in order to show a clear visual result. All the chromaticity coordinates are listed in Table 1. The highest color change was achieved for both FO and ND (Figure 5d) samples with a similar color change difference, followed by CFO 20 wt %. The change in color of the samples with 33 and 40 wt % of CFO is very subtle due to the high concentration and dark color of the CFO particles, which hinder the color effect of the TC particles.

**Table 1:** Chromaticity Coordinates of the Colored and Bleached States for Printed MC Inks

	chromaticity coordinates (*x*, *y*)	
sample	colored state	bleached state
40 wt % FO	0.41, 0.32	0.35, 0.34
40 wt % ND	0.39, 0.32	0.33, 0.35
40 wt % CFO	0.34, 0.33	0.32, 0.34
33 wt % CFO	0.33, 0.33	0.33, 0.34
20 wt % CFO	0.34, 0.34	0.33, 0.34

Figure 5e demonstrates that only ND and CFO could change the color of the composite at a 20 wt % content level for a maximum applied magnetic field of 50 mT. These results were obtained by gradually increasing the amplitude of the applied magnetic field from 0 to 50 mT until the color change was observed with the naked eye. All the tested particles changed the composite’s color at a 33 wt % content level, although FO required higher magnetic fields to induce this change. Notably, CFO required the least magnetic field to induce a color change in the composite at a 40 wt % content level, although the contrast is the lowest. However, by changing the TC particles to a more suitable color, it is possible to improve the MC visual effect. This superior magnetocaloric coupling performance of CFO is attributed to its higher magnetothermal properties or specific loss power (SLP) compared to the other magnetic structures used.^27^ While it is challenging to directly compare absolute SLP values across different studies and magnetic nanoparticles, the higher magnetothermal properties of CFO can be linked to factors such as the concentration of nanoparticles, their hydrodynamic and magnetic particle size, nanoparticle magnetization, the physical and chemical properties of the medium, and measurement conditions like magnetic field frequency and strength.^27,54^

As the composite material’s sensitivity to magnetic fields is enhanced by increasing the concentration of CFO particles, the color switching times (defined as the time required for the material to transition from its initial state (colored) to its final state (bleached or changed color) upon the application of an external stimulus, in this case an alternating magnetic field: 200 Oe, 100 kHz) were assessed as a function of CFO concentration under a 30 mT magnetic field, as depicted in Figure 5f. The increased ability to change color (indicated by shorter switching time) with higher CFO content suggests that a higher concentration of CFO nanoparticles in the composites does not significantly reduce the heating efficiency, despite decreasing the distance between nanoparticles.^55,56^ However, it is expected that if the CFO content increases further, the nanoparticles will be closer together, and dipole–dipole interactions might hinder the heating efficiency of the CFO particles.^56^

## 4 Conclusions

This study explored the influence of morphological and compositional properties of printed MC layers on their performance. Uniform particle distribution ensures consistent optical properties, while optimal layer thickness and minimal agglomeration enhance color change response and durability.

The developed MC inks exhibit viscosities ranging from 0.23 to 30 Pa·s, ideal for screen-printing, and show pseudoplastic behavior, with viscosity decreasing with increasing shear rate, ensuring efficient ink transfer. Mechanical tests on PVA substrates show variations in Young’s modulus due to the superior mechanical reinforcement of microsized ND, enhancing polymer–filler physical interactions and interface quality. Composites with higher nanoparticle content display improved thermal stability, indicated by delayed decomposition and slower weight loss.

Magnetic characterization reveals distinct behaviors: CFO-based composites exhibit coercivity of ≈0.24 mT and remanence of 1.35, 5.68, and 6.16 emu g^–1^, for CFO concentrations of 20, 33, and 40 wt %, respectively; ND composites show higher coercivity (0.68 mT) and remanence (13.69 emu g^–1^); and FO-based composites display superparamagnetic behavior without hysteresis, remanence, or coercivity. Only ND and CFO could change the composite color at 20 wt % content, while all particles did so at 33 wt %, with FO requiring higher magnetic fields to achieve the same effect. At 40 wt % content, CFO needed the least magnetic field for color change, attributed to its superior magnetothermal properties or SLP. Increased CFO content improves color change capability without significantly reducing heating efficiency, although further increases may hinder efficiency due to dipole–dipole interactions. These insights are essential for developing the next generation of high-performance, flexible MC materials.
